# Upregulation of miR-181c contributes to chemoresistance in pancreatic cancer by inactivating the Hippo signaling pathway

**DOI:** 10.18632/oncotarget.6298

**Published:** 2015-10-26

**Authors:** Meiyuan Chen, Min Wang, Simiao Xu, Xingjun Guo, Jianxin Jiang

**Affiliations:** ^1^ Department of Hepatic-Biliary-Pancreatic Surgery, Hubei Cancer Hospital, Wuhan, Hubei, 430079, China; ^2^ Department of Biliary-Pancreatic Surgery, Affiliated Tongji Hospital, Tongji Medical College, Huazhong University of Science and Technology, Wuhan, Hubei, 430030, China; ^3^ Department of Endocrinology and Metabolism, Tongji Hospital, Tongji Medical College, Huazhong University of Science and Technology, Wuhan, Hubei, 430030, China

**Keywords:** miR-181c, pancreatic cancer, Hippo signaling, chemoresistance

## Abstract

The Hippo signaling pathway plays a crucial role in regulating tissue homeostasis, organ size, tumorigenesis and cancer chemoresistance when deregulated. Physiologically, the Hippo core kinase cassette that consists of mamma-lian STE20-like protein kinase 1/2 (MST1/2), and large tumour suppressor 1/2 (LATS1/2), together with the adaptor proteins Salvador homologue 1 (SAV1) and MOB kinase activator 1 (MOB1), tightly restricts the activities of homologous oncoproteins Yes-associated protein (YAP) and transcriptional co-activator with PDZ-binding motif (TAZ) to low levels. However, how the Hippo kinase cassette core components are simultaneously inhibited, to exhibit constitutively inactivated Hippo signaling and activated YAP/TAZ in cancer remains puzzling. Herein, we reported that miR-181c directly repressed MST1, LATS2, MOB1 and SAV1 expression in human pancreatic cancer cells. Overexpression of miR-181c induced hyperactivation of the YAP/TAZ and enhanced expression of the Hippo signaling downstream genes *CTGF*, *BIRC5* and *BLC2L1*, leading to pancreatic cancer cell survival and chemoresistance *in vitro* and *in vivo*. Importantly, high miR-181c levels were significantly correlated with Hippo signaling inactivation in pancreatic cancer samples, and predicted a poor patient overall survival. These findings provide a novel mechanism for Hippo signaling inactivation in cancer, indicating not only a potentially pivotal role for miR-181c in the progression of pancreatic cancer, but also may represent a new therapeutic target and prognostic marker.

## INTRODUCTION

Pancreatic cancer is one of the most lethal gastrointestinal tract malignancies and the seventh most common cause of cancer-related death worldwide [1]. The overall prognosis for patients diagnosed with pancreatic cancer remains dismal, with 5-year survival rates averaging <5% [1]. This tumor is usually presented at advanced stage, when the surgical therapy is limited. Indeed, only 15–20% of patients are operable; for the majority of cases, the only therapeutic promise is cytostatic treatment [2]. Despite chemotherapy involving multiple drugs, including gemcitabine, the median survival time of patients with advanced pancreatic cancer over the past decades has been only about six months, mostly because of an almost complete resistance against chemotherapies [3, 4]. Hence, it would be of great clinical value to further understand the molecular mechanisms underlying pancreatic cancer chemoresistance and to identify effective early markers for the diagnosis and prognosis of the disease as well as novel therapeutic targets.

The Hippo signaling pathway is dysregulated in various human cancers and plays important roles in tumorigenesis as well as many other important biological processes [5–7]. Recently, accumulating evidence strongly suggested that the Hippo pathway may play important roles in chemotherapeutic drug resistance as well [8]. YAP and TAZ are the two major effectors and inhibited by Hippo pathway [9, 10]. Studies have consistently demonstrated that upregulation of YAP or TAZ rendered resistance of mammary and ovarian cancer cells to chemotherapeutic drugs taxol and cisplatin [11–15]. Moreover, hepatocellular carcinoma cells with high levels of YAP expression were doxorubicin-resistant, and hyper-activation of YAP was observed in 5-fluorouracil (5-FU)-resistant colon cancer cells and castration-resistant prostate tumor samples [16–18]. Additionally, downregulation of the Hippo pathway components mammalian STE20-like protein kinase 1/2 (MST1/2) and large tumour suppressor1/2 (LATS1/2) were observed in cancers, contributing to their resistance to diverse chemotherapeutic drugs [19–24]. Importantly, TAZ downregulation or MST1 upregulation were found to sensitize breast cancer and prostate cancer cells to taxol and cisplatin [15, 21]. Accordingly, the Hippo pathway is considered as an important regulator in cancer chemoresistance, and better understanding of the mechanisms that regulate Hippo pathway may provide new clues for more effective cancer therapy.

In the mammalian Hippo pathway, there are four core kinase cassette components: kinases MST1/2 and LATS1/2, as well as the adaptor proteins SAV1 and MOB1 [7]. Physiologically, the Hippo kinase cassette tightly balances YAP and TAZ activities, both spatially and temporally, to low levels through phosphorylation–ubiquitination mechanisms. When Hippo signaling is active, YAP and TAZ are phosphorylated by core complexes, then restricted to the cytoplasm and degraded [10, 25, 26]. Conversely, when Hippo signaling is absent, unphosphorylated YAP1/TAZ enter the nucleus and induce the transcriptional activity of TEA domain (TEAD) family members (TEAD1–TEAD4), by acting as the transcriptional co-activators [27–29]. In turn, the activated TEADs transcriptionally upregulate multiple downstream effectors to exert a pleiotropic role in tumor progression, including connective tissue growth factor (CTGF), baculoviral IAP repeat containing 5 (BIRC5) and BCL2-like 1 (BCL2L1), leading to the promotion of cell survival and chemoresistance [15, 30–32]. However, how MST1/2, LATS1/2, SAV1, and MOB1 are simultaneously repressed to exhibit constitutively activated YAP/TAZ in cancer remains unclear.

It has been well-established that microRNAs (miRNAs) are able to simultaneously repress a variety of target genes by binding to their mRNA 3′ untranslated regions (3′UTRs), and play important roles in tumorigenesis and malignant progression of human cancers [33, 34]. Herein, public microarray data and our results together suggested that miR-181c was substantially overexpressed in clinical pancreatic cancer samples and significantly correlated with poor prognosis. miR-181c directly repressed MST1, LATS2, MOB1 and SAV1, leading to YAP/TAZ activation and subsequent promotion of pancreatic cancer cell survival and chemoresistance *in vitro* and *in vivo*. Taken together, these findings uncover a novel regulatory mechanism for Hippo signaling inactivation, and might also provide a potential therapeutic target for pancreatic cancer.

## RESULTS

### Upregulation of miR-181c correlates with pancreatic cancer progression

**Figure 1:**
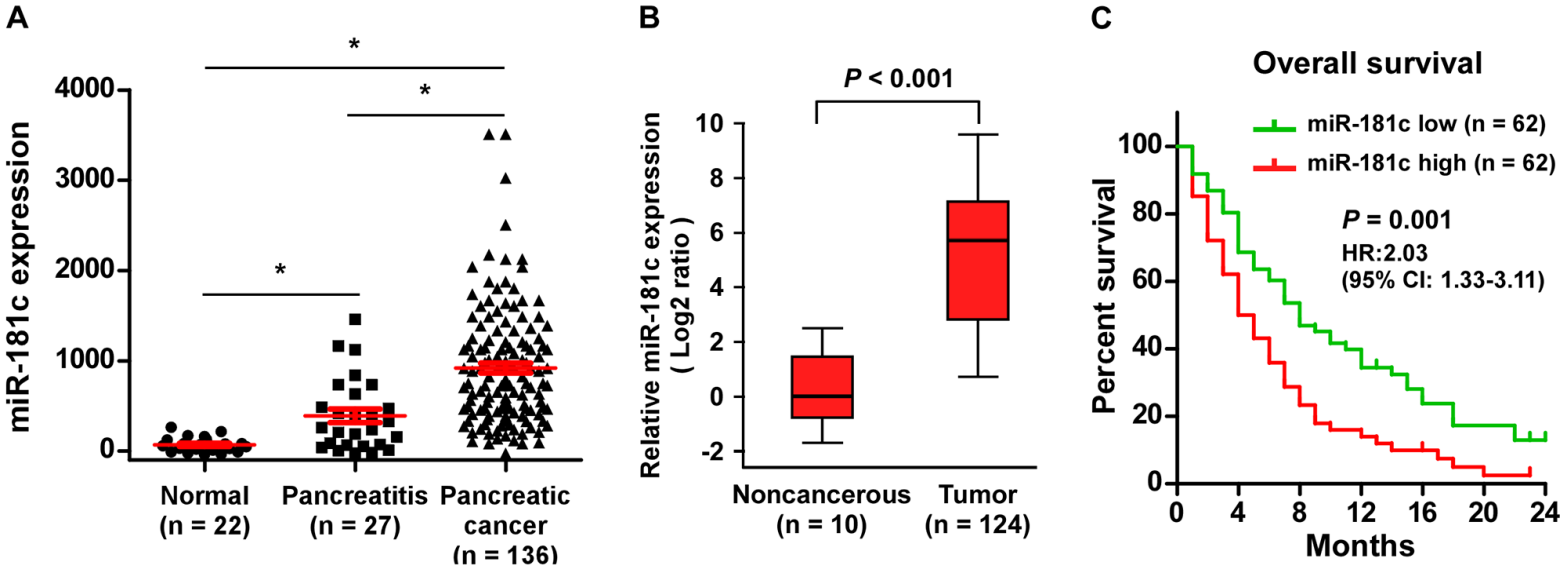
Upregulation of miR-181c correlates with pancreatic cancer progression **A.** miR-181c levels remained low in normal pancreatic tissues but became markedly higher in patients with pancreatitis and further elevated in pancreatic cancer patients assessed by analyzing a published microarray (GSE24279; Normal, *n* = 22; Pancreatitis, *n* = 27; Pancreatic cancer, *n* = 136). *P < 0.05. **B.** Real-time PCR analysis of miR-181c expression in 124 freshly collected pancreatic tissues compared to that in 10 non-cancerous pancreatic tissues. Transcript levels were normalized to *U6* expression. Boundaries of boxes represent lower and upper quartiles, respectively. Lines within boxes and whiskers denote median and extremum, respectively. *P* < 0.001, 2-tailed Student's *t*-test. **C.** Kaplan–Meier analysis of 2-year overall survival curves of patients with pancreatic cancer with high miR-181c expression (>median, *n* = 62) versus low miR-181c expression (<median, *n* = 62) [hazard ratio (HR) = 2.03; 95% confidence interval (CI): 1.33–3.11]. *P* < 0.001, log-rank test.

Analysis using a published microarray (NCBI/GEO/GSE24279; *n* = 185, including 22 normal, 27 pancreatitis and 136 pancreatic cancer samples), we found that miR-181c levels remained low in normal pancreatic tissues but became markedly higher in patients with pancreatitis and further elevated in pancreatic cancer patients (Figure 1A). We further confirmed miR-181c expression in pancreatic cancer tissues using real-time PCR. As shown in Figure 1B, miR-181c expression was markedly increased in 124 pancreatic cancer samples compared with that in 10 non-cancerous pancreatic tissues. In the pancreatic cancer samples, statistical analysis revealed that increased miR-181c expression strongly correlated with TNM stage and histological differentiation (all *P* < 0.05) (Supplementary Table 1 and 2). Importantly, patients with high miR-181c expression had a significantly poorer overall survival compared to patients with low miR-181c expression (*P* = 0.001; hazard ratio = 2.03, 95% CI = 1.33–3.11; Figure 1C). Thus, these results suggest that upregulation of miR-181c might be involved in human pancreatic cancer progression.

### MiR-181c directly targets the core components of the Hippo signaling pathway

**Figure 2:**
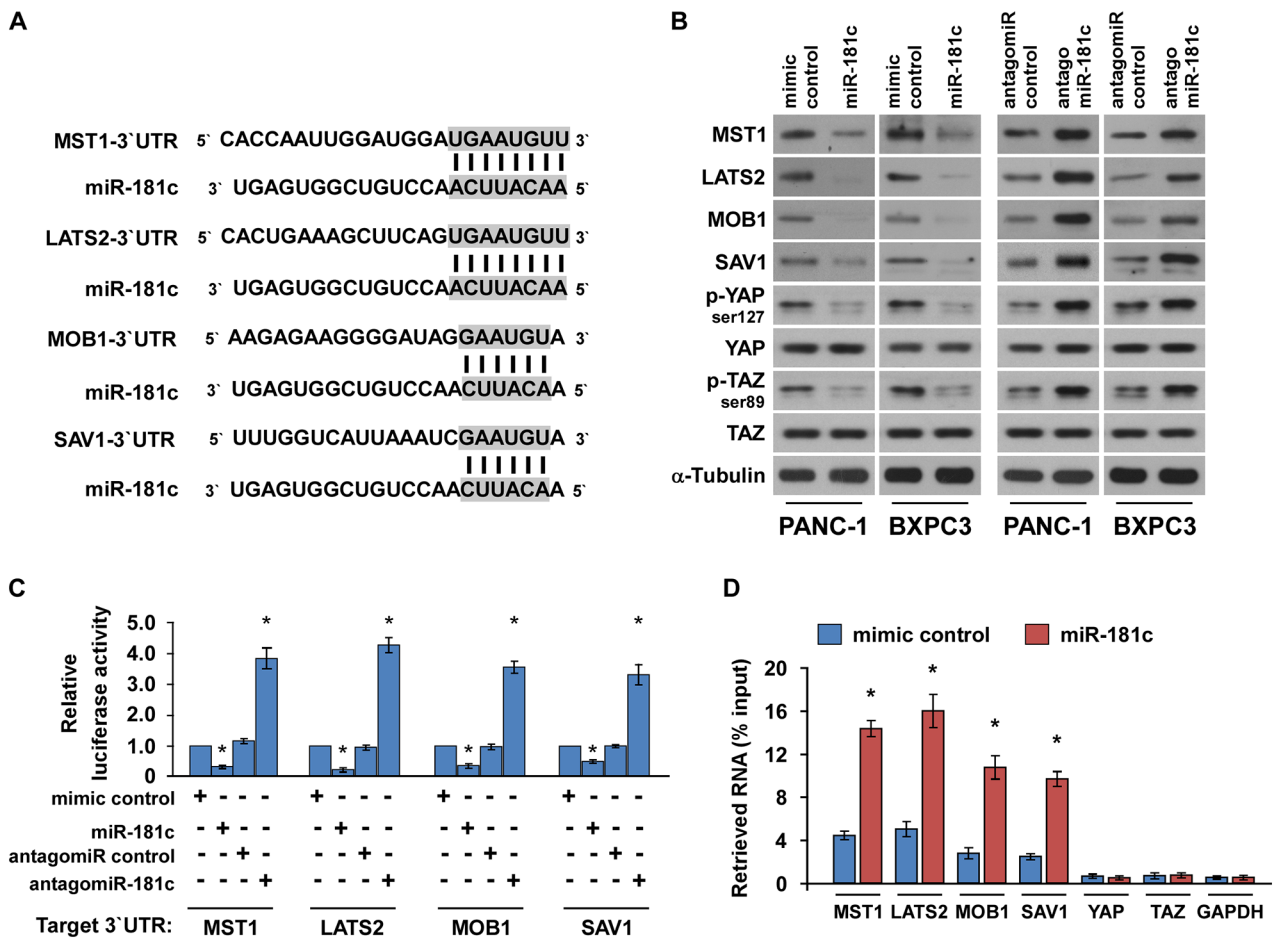
MiR-181c directly targets the core components of the Hippo signaling pathway **A.** Predicted miR-181c target sequence in 3′UTRs of *MST1*, *LATS2*, *MOB1* and *SAV1*. **B.** Western blots of MST1, LATS2, MOB1 and SAV1 expression. α-Tubulin served as the loading control. **C.** Luciferase assay of cells transfected with pGL3-MST1-3′UTR, pGL3-LATS2-3′UTR, pGL3-MOB1-3′UTR or pGL3-SAV1-3′UTR reporter with miR-181c mimic, antagomiR-181c, mimic control or antagomiR control. **D.** MiRNP IP assay showing the association between miR-181c and *MST1*, *LATS2*, *MOB1* and *SAV1* transcripts, but not *YAP* or *TAZ* in PANC-1 cells. *GAPDH* served as the negative control. Error bars represent the mean ± s.d. of three independent experiments. *P < 0.05.

Recent studies indicated that the Hippo signaling is deregulated and plays an important role in the development and progression of pancreatic cancer [35–37]. Interestingly, using the publicly available algorithms TargetScan and miRanda, we found that the core components of the Hippo signaling pathway, i.e., *MST1*, *LATS2*, *MOB1* and *SAV1* might be potential targets of miR-181c (Figure 2A). We exogenously overexpressed miR-181c via miR-181c mimic transfection, and endogeneously silencing miR-181c by transfecting antagomiR-181c (Supplementary Figure 1). Western blotting analysis revealed that miR-181c overexpression significantly suppressed expression levels of MST1, LATS2, MOB1 and SAV1, and phosphorylation levels of downstream effectors YAP (p-YAP^ser127^) and TAZ (p-TAZ^ser89^). In contrast, miR-181c inhibition increased them, suggesting that miR-181c negatively regulated these proteins (Figure 2B). Furthermore, luciferase assay showed that miR-181c overexpression attenuated, while inhibition of miR-181c elevated the reporter activities driven by the 3′UTRs of these transcripts (Figure 2C). However, ectopic expression of the miR-181c did not exhibit repressive effects on the reporter activities driven by the mutant 3′UTRs of these transcripts within miR-181c–binding seed regions (Supplementary Figure 2A). Moreover, microribonucleoprotein (miRNP) immunoprecipitation (IP) assay revealed a selective association of miR-181c with *MST1*, *LATS2*, *MOB1* and *SAV1*, but not with *YAP*, *TAZ* or *GAPDH* (Figure 2D), further indicating the specific effects of miR-181c on these targets. In addition, individual overexpression of these targets potently inhibited the TEAD activity in miR-181c-overexpressing cells (Supplementary Figure 2B), demonstrating that MST1, LATS2, MOB1 and SAV1 were functional effectors of miR-181c on regulating TEAD activation. Collectively, our results suggest that miR-181c directly targets MST1, LATS2, MOB1 and SAV1.

### MiR-181c inactivates the tumor-suppressive Hippo signaling pathway

**Figure 3:**
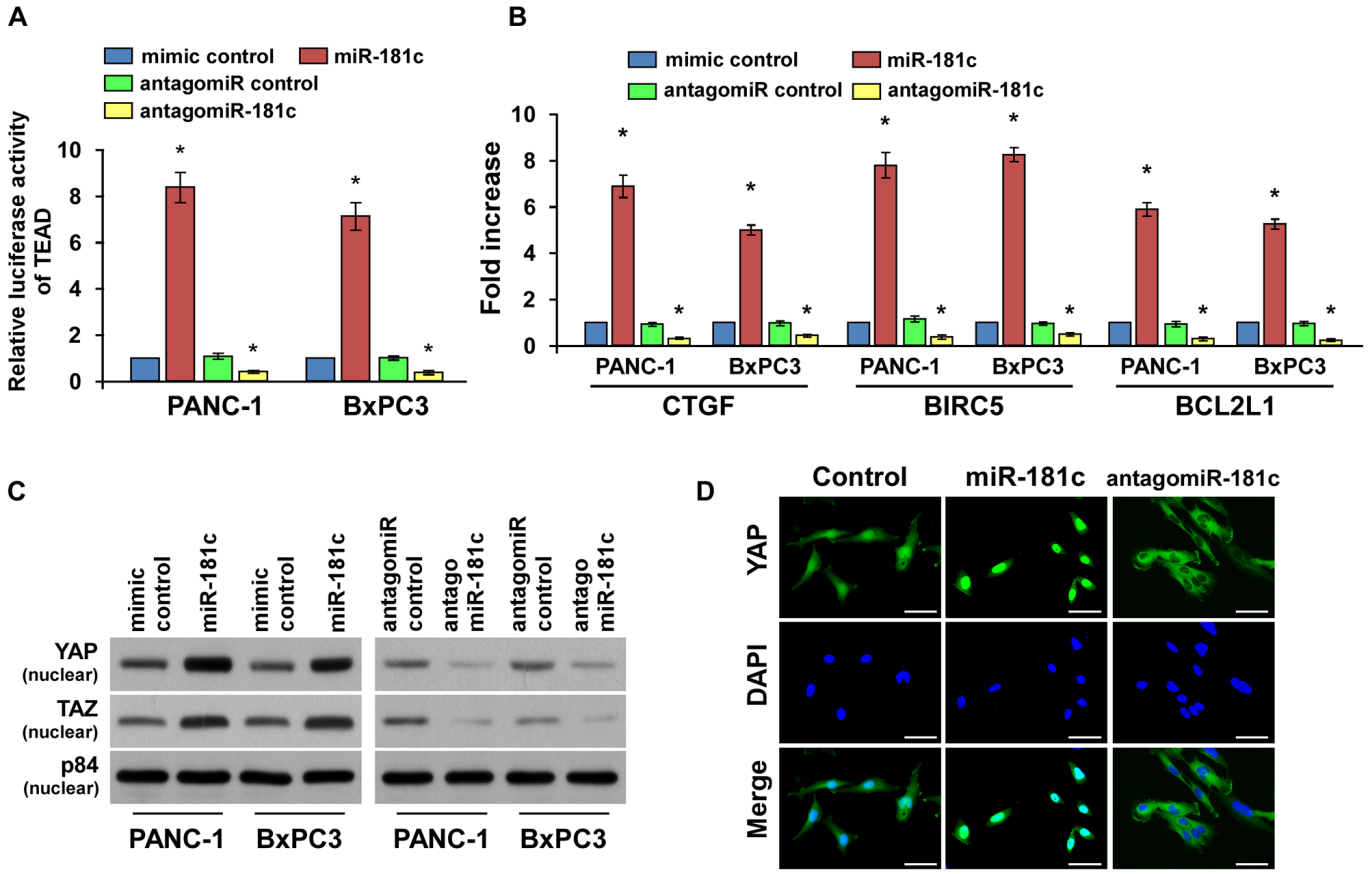
MiR-181c inactivates the tumor-suppressive Hippo signaling pathway **A.** TEAD transcriptional activity was assessed in the indicated cells by TEAD-dependent, promoter-driven firefly luciferase reporter construct. **B.** Real-time PCR analysis of *CTGF*, *BIRC5* and *BCL2L1* in indicated cells. Transcript levels were normalized to *GAPDH* expression. **C.** Western blotting of nuclear YAP and TAZ expression. The nuclear protein p84 was used as the nuclear protein marker. **D.** Fluorescent immunostaining of YAP in the control, miR-181c-overexpressing and miR-181c-silenced PANC-1 cells. Scale bars: 10 μm. Error bars represent the mean ± s.d. of three independent experiments. *P < 0.05.

The tumor-suppressive Hippo signaling pathway was identified as a major regulator of cellular apoptosis by the nuclear translocation of YAP and TAZ and interaction with TEAD transcription factors. We further examined the role of miR-181c in Hippo signaling pathway. As shown in Figure 3A and 3B, we found that miR-181c overexpression in pancreatic cancer cells significantly increased, but silencing of miR-181c reduced TEAD-dependent luciferase activity, and expression levels of the Hippo downstream genes *CTGF*, *BIRC5* and *BCL2L1* in pancreatic cancer cells. In addition, overexpression of miR-181c increased, while silencing miR-181c reduced the binding capability of TEAD1 with CTGF promoter (Supplementary Figure 3). Moreover, cellular fractionation and fluorescent immunostaining assays revealed that overexpression of miR-181c increased nuclear accumulation of YAP and TAZ, while silencing of miR-181c reduced nuclear YAP and TAZ expression (Figure 3C and 3D). Therefore, our results suggest that miR-181c inactivates the Hippo signaling pathway.

### MiR-181c promotes chemoresistance in pancreatic cancer *in vitro*

**Figure 4:**
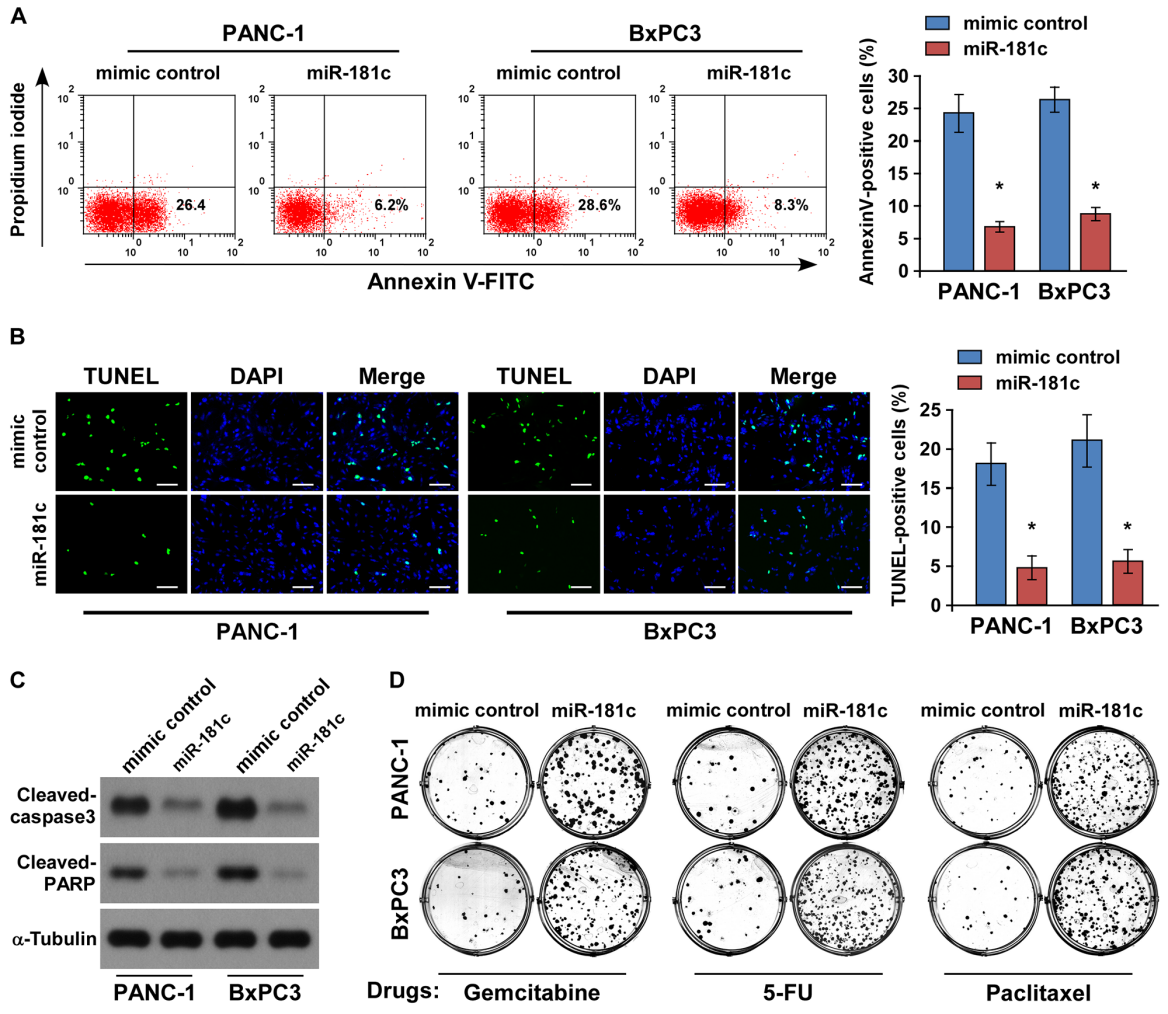
Upregulation of miR-181c promotes pancreatic cancer cell chemoresistance *in vitro* **A.** Annexin V-FITC/PI staining of indicated cells treated with gemcitabine (5 μM) for 24 h. **B.** Representative micrographs (left) and quantification (right) of TUNEL-positive cells following 36-h gemcitabine (5 μM) treatment. Scale bars: 50 μm. **C.** Western blotting of cleaved caspase3 and PARP expression. α-Tubulin was used as the loading control. **D.** Representative micrographs of crystal violet–stained PANC-1 and BxPC3 pancreatic cancer cell colonies in the presence of gemcitabine (5 μM), 5-FU (5 μM), or paclitaxel (10 μM). Error bars represent the mean ± s.d. of three independent experiments. *P < 0.05.

Since resistance of pancreatic cancer cells to chemotherapeutic drugs is crucial for the inefficient therapy, and recent evidence strongly suggests that Hippo pathway inactivation plays important roles in chemotherapeutic drug resistance [8, 11–15], we then investigated the role of miR-181c upregulation in the drug-resistant survival. Annexin V and TUNEL assays demonstrated that ectopic expression of miR-181c reduced the apoptosis rate of PANC-1 and BxPC3 pancreatic cancer cells by resisting to the chemotherapeutic agent gemcitabine (Figure 4A and 4B). Meanwhile, gemcitabine treatment had no effect on miR-181c expression in pancreatic cancer cells (Supplementary Figure 4A). Moreover, the effect of miR-181c on apoptotic protection was confirmed by examining the cleavages of pro-caspase 3 and poly (ADP-ribose) polymerase (PARP) in pancreatic cancer cells. As shown in Figure 4C, cleavages of both caspase 3 and PARP were suppressed in the miR-181c-overexpressing cells treated with gemcitabine. Importantly, the colony formation assay indicated that overexpression of miR-181c rendered resistance of pancreatic cancer cells in the presence of gemcitabine, 5-FU, or paclitaxel to form many more colonies as compared to the control (Figure 4D and Supplementary Figure 4B).

**Figure 5:**
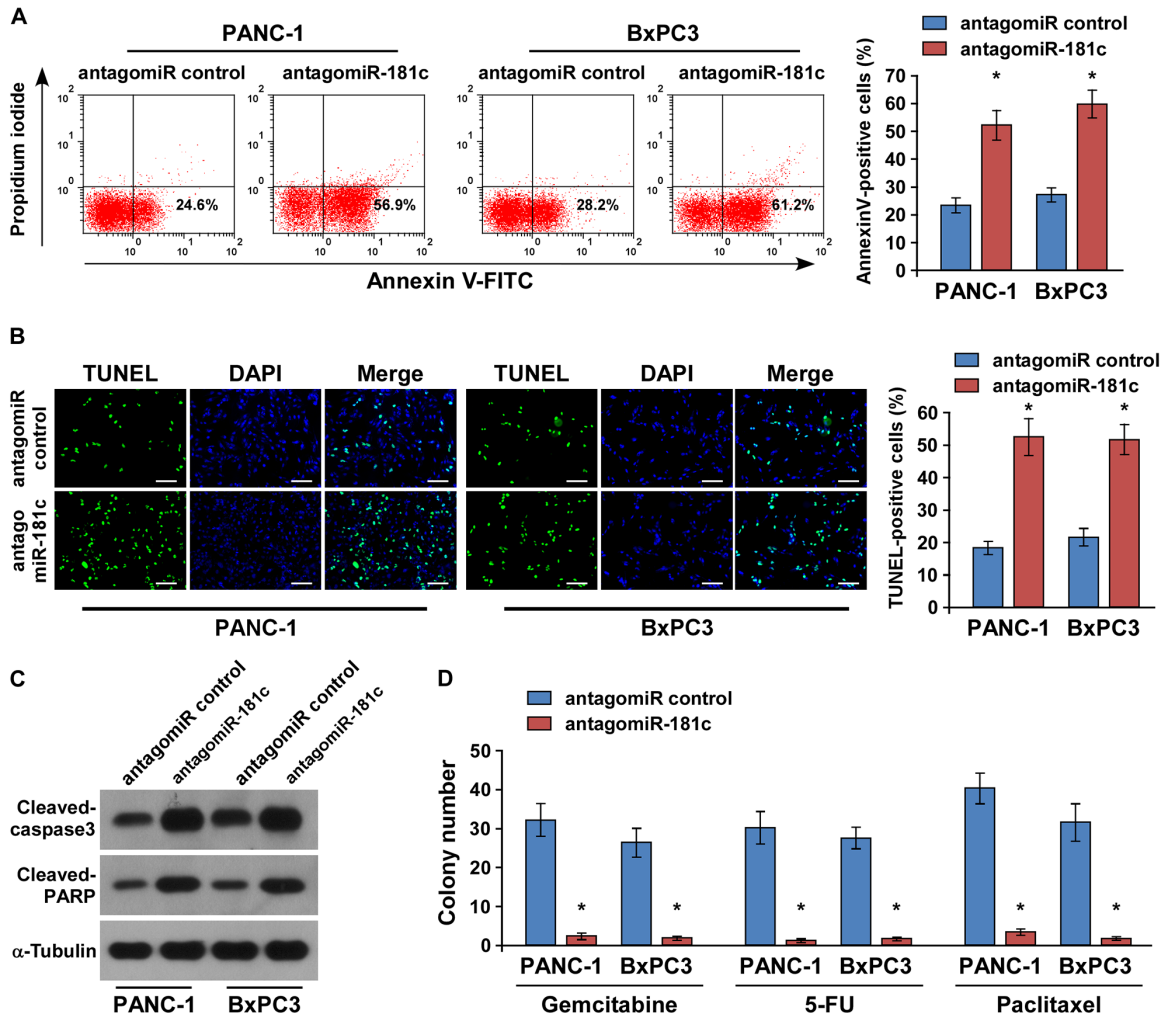
Silencing of miR-181c increases chemotherapeutic drug–induced apoptosis of pancreatic cancer cells *in vitro* **A.** Cells were treated with gemcitabine (5 μM) for 24 h and stained with annexin V–FITC/PI. **B.** Silencing of miR-181c increased the number of TUNEL-positive cells following 36-h treatment with gemcitabine (5 μM). Scale bars: 50 μm. **C.** Western blotting of cleaved caspase3 and PARP expression. α-Tubulin was used as the loading control. **D.** Quantification of crystal violet–stained PANC-1 and BxPC3 pancreatic cancer cell colonies in the presence of gemcitabine (5 μM), 5-FU (5 μM), or paclitaxel (10 μM). Error bars represent the mean ± s.d. of three independent experiments. *P < 0.05.

Conversely, the role of miR-181c in pancreatic chemoresistance was further examined by endogenously silencing miR-181c. As shown in Figure 5A and 5B, we found that silencing of miR-181c increased the apoptosis rate of PANC-1 and BxPC3 pancreatic cells under the treatment of gemcitabine. Moreover, we also found that neither overexpression of miR-181c nor silencing of miR-181c had an effect on the apoptotic percentage of PANC-1 and BxPC3 cell lines without chemo-drug treatment, suggesting that miR-181c exerted its effects on pancreatic cancer cell survival in the presence of gemcitabine (Supplementary Figure 4C and 4D). Consistently, cleavages of both caspase 3 and PARP were increased in the miR-181c–silenced cells treated with gemcitabine (Figure 5C). Moreover, gemcitabine, 5-FU, and paclitaxel treatment significantly reduced the number of colonies formed by the miR-181c–silenced pancreatic cancer cells. Collectively, these results suggest that miR-181c promotes pancreatic cancer cell resistance to chemotherapeutic drugs *in vitro*.

### Inhibition of miR-181c sensitizes pancreatic cancer cells to chemotherapeutic drugs *in vivo*

**Figure 6:**
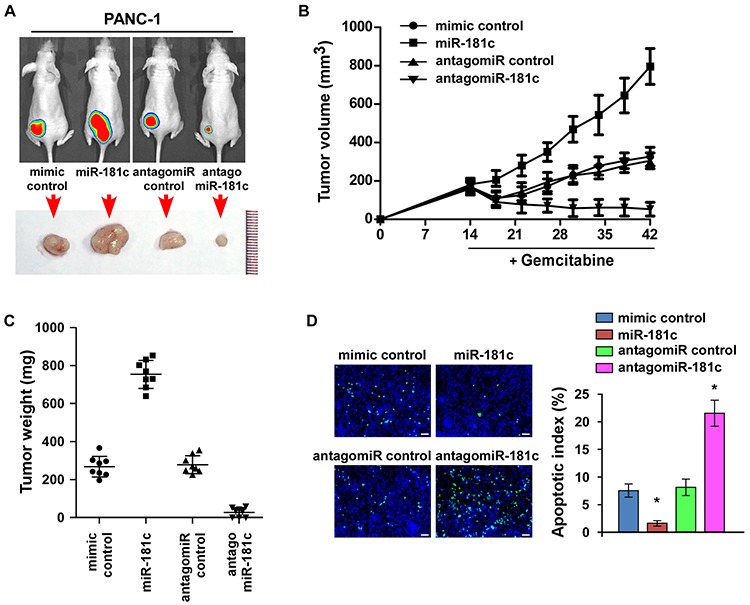
Inhibition of miR-181c sensitizes pancreatic cancer cells to chemotherapeutic drugs *in vivo* **A.** Xenograft model in nude mice. Representative images of tumor-bearing mice (top) and tumors from each experimental group (bottom). **B.** Tumor volumes of tumors in the miR-181c–overexpressing, miR-181c–silenced, and control groups were measured on indicated days. Data presented are the mean ± s.d. **C.** Tumor weights of each group. **D.** Apoptotic index (right) was determined using the percentage of TUNEL-positive cells (left). Scale bars: 50 μm. Error bars represent the mean ± s.d. of three independent experiments. *P < 0.05.

The promotive effect of miR-181c on pancreatic cancer cell chemoresistance was further examined *in vivo*. Mice were inoculated subcutaneously (5 × 10^6^ PANC-1 cells per mouse) in the left dorsal flank. Two weeks later, the mice were randomly divided into four groups (*n* = 8/group). Each group of mice were intratumorally injected with 200 μg mimic control, miR-181c mimic, antagomir control, or antagomiR-181c (diluted in phosphate-buffered saline [PBS] at 2 mg/ml) three times per week for four weeks, combined with intraperitoneal injection of gemcitabine (50 mg/kg) weekly (Figure 6A and Supplementary Figure 5). The tumor volumes and weight were increased in the miR-181c plus gemitabine group, but were decreased in the antagomiR-181c plus gemcitabine group, as compared to the controls, respectively (Figure 6A–6C). Significantly, the combined antagomiR-181c and gemcitabine treatment markedly restricted the tumor growth to low volumes (Figure 6C). These results suggested that miR-181c promoted gemcitabine resistance and tumor growth, while injection of antagomiR-181c dramatically sensitized PANC-1 cells to gemcitabine treatment and inhibited tumor growth. Meanwhile, tumors injected with miR-181c mimic had decreased TUNEL-positive apoptotic cells, whereas tumors injected with antagomir-181c had a higher percentage of TUNEL-positive apoptotic cells (Figure 6D). Taken together, these findings suggest that miR-181c upregulation promotes pancreatic cancer cell chemoresistance, and inhibition of miR-181c sensitizes pancreatic cancer cells to gemcitabine *in vivo*.

### YAP/TAZ activation is essential for miR-181c-induced chemoresistance

**Figure 7:**
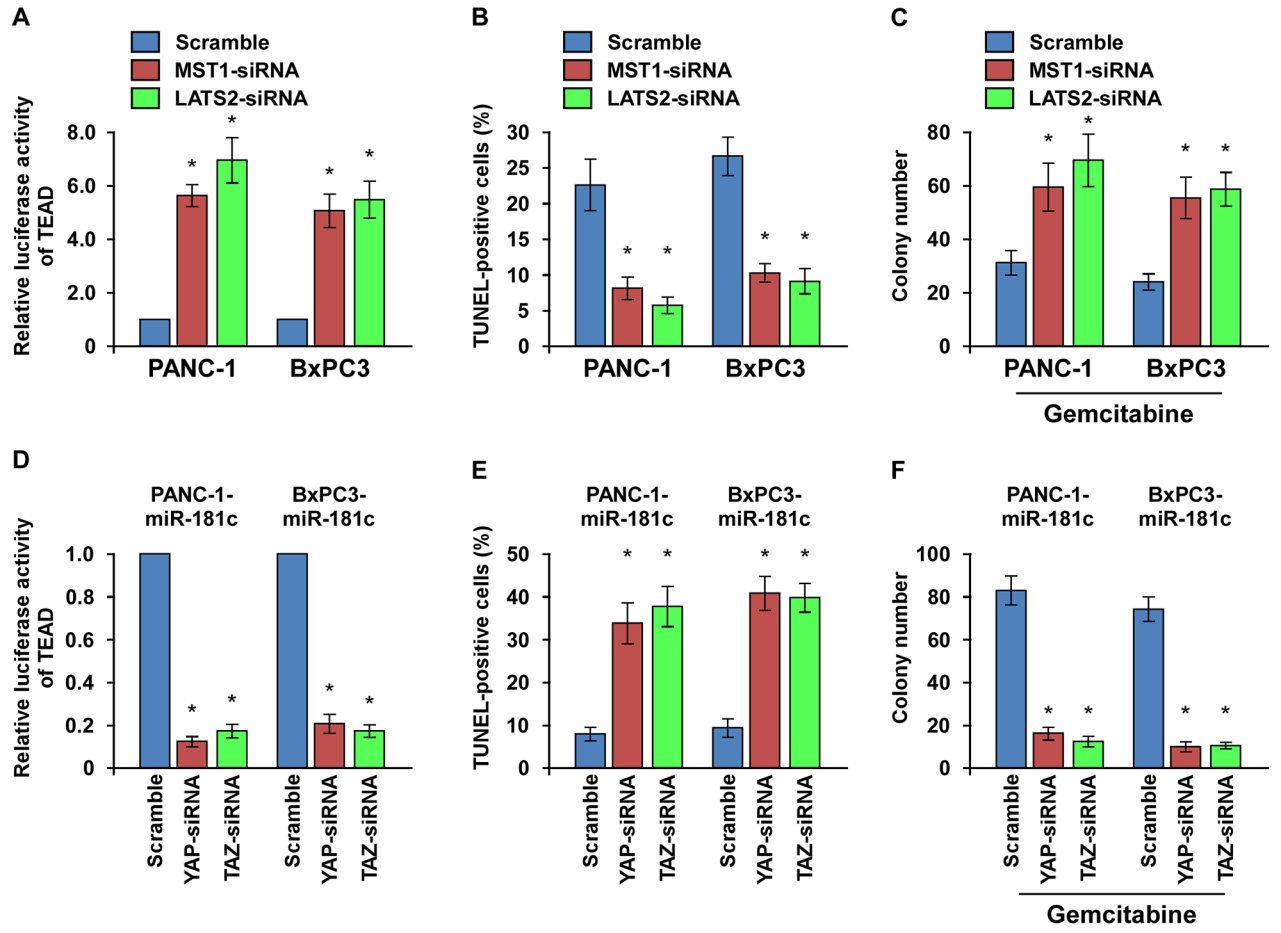
YAP/TAZ activation is essential for miR-181c-induced chemoresistance **A.** TEAD transcriptional activity was increased by MST1 or LATS2 depletion in the cells. (B and C) TUNEL **B.** and colony formation assays **C.** indicating that silencing of MST1 or LATS2 promoted pancreatic cancer cell chemoresistance and survival. **D.** TEAD transcriptional activity induced by miR-181c was suppressed by YAP or TAZ depletion in the cells. (E and F) TUNEL **E.** and colony formation assays **F.** indicating that YAP or TAZ silencing abrogated miR-181c–induced promotion of pancreatic cancer cell chemoresistance and survival. Error bars represent the mean ± s.d. of three independent experiments. *P < 0.05.

We then explored the functional significance of Hippo signaling in the chemoresistance of pancreatic cancer cells by silencing of the two key kinases MST1 and LATS2. As shown in Figure 7A and Supplementary Figure 6A, either silencing of MST1 or LATS2 increased the TEAD activity. MST1 or LATS2 silencing protected the apotosis induced by gemcitabine, and rendered pancreatic cancer cells to form more colonies in the presence of gemcitabine (Figure 7B and 7C). Thus, these results suggest that inactivation of Hippo signaling plays an important role in the chemoresistance in pancreatic cancer.

Furthermore, we investigated whether activation of the downstream effectors YAP/TAZ is essential for miR-181c-induced chemoresistance. As expected, the stimulatory effect of miR-181c on TEAD luciferase activity and expression of CTGF, BIRC5 and BCL2L1 were inhibited by YAP or TAZ silencing (Figure 7D and Supplementary Figure 6B, 6C). Moreover, YAP or TAZ silencing abrogated the promotive effects, but overexpression of YAP or TAZ rescued the repressive effects of miR-181c on gemcitabine resistance, as indicated by the TUNEL and colony formation assays (Figure 7E, 7F and Supplementary Figure 6D). Collectively, these results indicate that activation of YAP/TAZ is critical for miR-181c–induced chemoresistance in pancreatic cancer cells.

### Clinical relevance of upregulation of miR-181c– mediated Hippo signaling inactivation in pancreatic cancer

**Figure 8:**
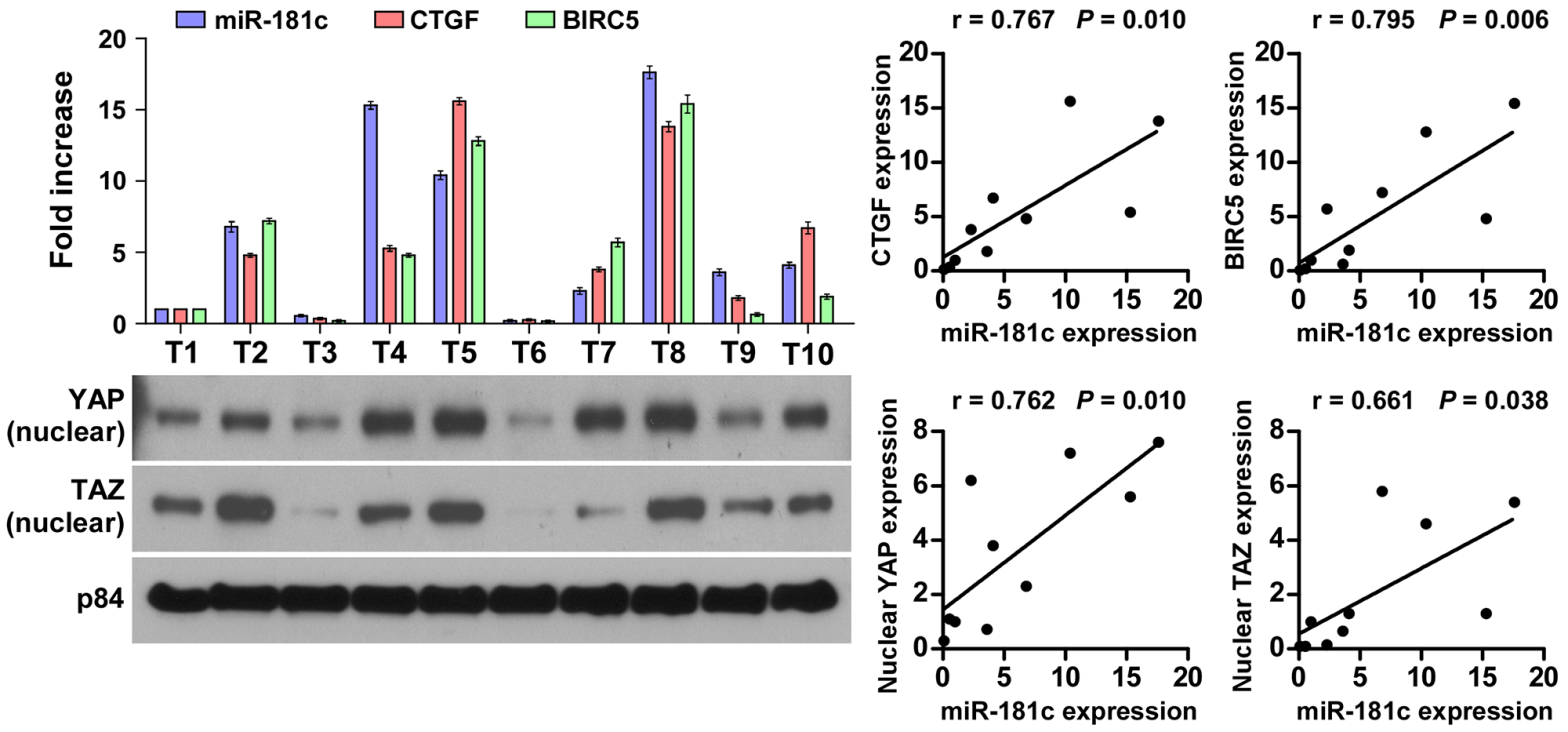
Clinical relevance of upregulation of miR-181c–mediated Hippo signaling inactivation in pancreatic cancer Expression analysis (left top) and correlation (right) of miR-181c expression and *CTGF* and *BIRC5* mRNA levels, as well as (left bottom) nuclear YAP and TAZ protein expression in 10 freshly collected human pancreatic cancer tissue samples (T). The ratio of first sample (nuclear YAP/α-tubulin and nuclear TAZ/α-tubulin) was considered as 1.0. α-Tubulin was used as the loading control.

Finally, we examined whether miR-181c–mediated inactivation of Hippo signaling in pancreatic cancer was clinically relevant. As shown in Figure 8, miR-181c levels in 10 freshly collected pancreatic cancer samples were significantly positively correlated with mRNA levels of the Hippo downstream genes *CTGF* (*r* = 0.767, *P* = 0.010) and *BIRC5* (*r* = 0.795, *P* = 0.006), as well as nuclear expression levels of YAP (*r* = 0.762, *P* = 0.010) and TAZ (*r* = 0.661, *P* = 0.038). Consistently, IHC staining results revealed that YAP/TAZ was strongly expressed in pancreatic cancer cells but not in the surrounding stromal cells, and nuclear YAP/TAZ expression correlated with miR-181c expression (Supplementary Figure 7). Collectively, these results further support the notion that upregulation of miR-181c inactivates the Hippo signaling, and results in poor clinical outcome in pancreatic cancer.

## DISCUSSION

The YAP and TAZ transcriptional co-factors are two key downstream effectors of the Hippo signaling pathway, and exert pleiotropic roles in both physiological and pathological processes, such as organ size control, tissue regeneration, differentiation, stem cell renewal, and tumorigenesis [6, 9, 10]. Under physiological conditions, YAP and TAZ are largely restricted to a low level of activities by the Hippo kinase cascade consisting of MST1, LATS2, MOB1 and SAV1, which sequentially phosphorylate YAP/TAZ to restrict in cytoplasm and degradate [10, 25, 26]. In contrast, mounting evidence has indicated that YAP and TAZ are constitutively activated in a wide range of tumor types and are involved in tumor progression by promoting uncontrolled cell proliferation, resistance to apoptosis and even distant metastasis [7, 38]. However, how cancer cells override the negative regulation induced by MST1, LATS2, MOB1, and SAV1 to exhibit constitutively inactivated Hippo signaling and activated YAP/TAZ remains puzzling. Herein, we demonstrated that miR-181c was substantially overexpressed in pancreatic cancer and induced hyper-activation of YAP/TAZ via directly targeting MST1, LATS2, SAV1 and MOB1. Therefore, our findings represent a novel mechanism that simultaneously disrupts the negative regulation by the core kinase cassette, leading to constitutive YAP/TAZ activation in cancer cells.

The prognosis of pancreatic cancer is rather poor, partly because it is frequently diagnosed at an advanced stage precluding surgical resection, and partly because it is characterized by a chemoresistant phenotype. Acting as a nucleoside analog drug, gemcitabine is now one of the first-line chemotherapeutic drugs in pancreatic cancer by inducing faults and damage in DNA replication. However, gemcitabine-resistance was observed in part of the pancreatic cancer patients, suggesting that there might be some other mechanisms responsible for the survival of pancreatic cancer cells. Although different mechanisms, including ineffective metabolic drug conversion, abnormal membrane receptor transport, increased DNA repair, and apoptotic pathway alteration have been identified, the molecular mechanism of pancreatic cancer cell chemoresistance remains poorly understood. Recent evidence indicated that Hippo signaling inactivation and YAP/TAZ hyperactivation played important roles in chemotherapeutic drug resistance, rendering different cancers insensitive to drug-induced apoptosis, suggesting that Hippo signaling might be a novel target for cancer chemotherapy [8, 11–15]. Our results revealed that miR-181c upregulation promoted pancreatic cancer cell chemoresistance by inactivating Hippo signaling and subsequently activating YAP/TAZ. In contrast, silencing of miR-181c upregulated MST1, LATS2, SAV1 and MOB1, and reconstituted the Hippo signaling, leading to increased sensitivity to drug-induced apoptosis. More importantly, the delivery of antagomiR-181c dramatically sensitized pancreatic cancer cells to gemcitabine treatment *in vivo*, suggesting an effective role of antagomiR-181c in pancreatic cancer chemotherapy. Taken together, our results not only suggest miR-181c–induced Hippo signaling inactivation as a novel mechanism for pancreatic cancer chemoresistance; but also propose that miR-181c might be a potential therapeutic target for human pancreatic cancer.

Recent evidence has revealed that Hippo signaling pathway inactivation plays an important role in the development of human cancers [7]. In fact, many of the genes involved in the Hippo signaling pathway are recognized as tumor suppressors, while *YAP*/*TAZ* is identified as an oncogene. Transgenic mice with liver-specific YAP overexpression exhibit dramatically increased liver size and eventually develop tumors, while LATS1 knockout leads to soft tissue sarcoma and ovarian tumor development [39–41]. Consistently, amplification of YAP or loss-of-function mutation of Hippo pathway components such as *MOB1* and *SAV1* have been identified in a series of human cancers [14, 42, 43]. Notably, upregulated YAP expression is also found in human pancreatic cancer, and ectopic YAP expression transforms non-tumorigenic human pancreatic epithelial cells and drives recurrence and progression of pancreatic cancer, suggesting that Hippo signaling might be involved in pancreatic cancer development [36, 37]. Interestingly, using published microarray–based high-throughput assessment, we found that miR-181c expression was markedly higher in patients with pancreatitis, which was an early event of pancreatic cancer development. Moreover, miR-181c inactivated the Hippo signaling and induced YAP activation. Thus, our findings suggest that miR-181c might be involved in the development of pancreatic cancer by inactivating the Hippo signaling. However, the biological role of miR-181c in pancreatitis and its relationship with pancreatic cancer development in cirrhotic tissues require further investigation.

MiR-181c has been found to be upregulated in multiple human cancers, and upregulation of miR-181c contributes to cancer cell proliferation, migration and invasion via different mechanisms [44–47]. However, the expression of miR-181c has also been shown to be downregualted in glioblastoma multiforme, and predicted a poor patient survival [48]. These findings indicate that miR-181c functions as both an oncomir and tumor-suppressive miRNA, depending on the tumor type. To investigate the clinical significance and the precise mechanism of action of miR-181c in pancreatic cancer pathogenesis, we examined miR-181c expression in pancreatic cancer samples and found that miR-181c was markedly upregulated in pancreatic cancer tissues as compared to normal tissues. Upregulation of miR-181c significantly correlated with the clinicopathological features and poorer overall survival of pancreatic cancer patients, suggesting that miR-181c might be associated with the progression of pancreatic cancer. Consistently, miR-181c dramatically promoted pancreatic cancer cell chemoresistance by inactivating the Hippo signaling pathway, further demonstrating the tumor-promoting role of miR-181c in pancreatic cancer. Of note, by analysis of the promoter region of miR-181c using the CONSITE program, we found two typical response elements of the transcriptional factor nuclear factor κB (NF-κB), which is hyperactivated in pancreatic cancer [49]. Meanwhile, the *miR-181c* locus is located in a region within 19p13 reported to be amplified in different human cancers [47, 50], suggesting that miR-181c overexpression in pancreatic cancer might be associated with genomic amplification. Thus, it would be of great interest to further investigate whether upregulation of miR-181c in pancreatic is attributed to genomic amplification and/or NF-κB-mediated transcriptional upregulation.

In summary, our study has revealed that miR-181c upregulation plays an important role in pancreatic cancer progression and miR-181c is a critical repressor of Hippo signaling by targeting the core kinase cassette, i.e., MST1, LATS2, SAV1 and MOB1. Understanding the precise role of miR-181c in pancreatic cancer pathogenesis and in the Hippo signaling pathway promises to increase our knowledge of the biological basis of cancer development and may also facilitate the development of new therapeutic strategies against pancreatic cancer.

## MATERIALS AND METHODS

### Cells

The pancreatic cancer cell lines PANC-1 and BXPC3 were maintained in RPMI 1640 (Invitrogen, Carlsbad, CA, USA) supplemented with 10% FBS (HyClone, Logan, UT, USA).

### Patient information and tissue specimens

The 124 paraffin-embedded, archived pancreatic cancer samples used in this study were histopathologically and clinically diagnosed at the Hubei Cancer Hospital between 2008 and 2011. Clinical staging and clinicopathological TNM classification were determined according to the criteria proposed by International Union Against Cancer (UICC) criteria. Written informed consent was obtained from all patients prior to the study. The Institutional Research Ethics Committee approved the use of the clinical specimens for research purposes. The clinicopathological characteristics of the samples are summarized in Supplementary Table 1. Freshly collected pancreatic cancer tissue specimens were each collected from 10 patients, and were frozen and stored in liquid nitrogen until used.

### Plasmid, oligonucleotides, and transfection

The TEAD Luciferase Reporting system was purchased from lifeome (Oceanside, CA, USA). The 3′UTR regions of human MST1, LATS2, MOB1 and SAV1, generated by PCR amplification from PANC-1 pancreatic cancer cell line, were cloned into the pGL3 luciferase reporter plasmid (Promega, Madison, WI). We purchased miR-181c mimic, miR-181c antagonist (antagomiR-129-5p), and controls from RiboBio (Guangzhou, China). Transfection of the plasmids, siRNAs, miR-181c mimic, and antagomiR-181c were performed using Lipofectamine 2000 (Invitrogen) according to the manufacturer's instructions.

### Western blotting analysis

Cells were harvested in cell lysis buffer (Cell Signaling Technology, Danvers, MA, USA) and heated for 5 min at 100°C. Equal quantities of denatured protein samples were resolved on 10% SDS-polyacrylamide gels, and then transferred onto PVDF membranes (Roche, Basel, Switzerland). After blocking with 5% non-fat dry milk in TBS/0.05% Tween 20, membranes were incubated with a specific primary antibody, followed by a horseradish peroxidase–conjugated secondary antibody. Proteins were visualised using ECL reagents (Pierce, Rockford, IL, USA). Antibodies against MST1, LATS2, MOB1, SAV1, YAP, TAZ and p84 were purchased from Abcam (Cambridge, MA, USA). The membranes were stripped and reprobed with an anti–α-tubulin antibody (Sigma-Aldrich, St. Louis, MO, USA) as the loading control.

### MiRNA extraction and real-time quantitative PCR

Total miRNA from cultured cells and fresh surgical pancreatic tissues was extracted using a mirVana miRNA Isolation Kit (Ambion, Austin, TX, USA) according to the manufacturer's instructions. We synthesised cDNA from 10 ng total RNA using a TaqMan miRNA reverse transcription kit (Applied Biosystems, Foster City, CA, USA), and quantified the expression levels of miR-181c using a miRNA-specific TaqMan MiRNA Assay Kit (Applied Biosystems). The expression of miRNA was defined based on the Ct, and relative expression levels were calculated as 2^−[(Ct of miR-181c) – (Ct of U6)]^ after normalisation with reference to expression of U6 small nuclear RNA.

### Terminal deoxynucleotidyl transferasedUTP nick end labeling (TUNEL) assay

Apoptotic DNA fragmentation was examined using an *in situ* DeadEnd™ Fluorometric TUNEL System assay kit (Promega, Madison, WI, USA) according to the manufacturer's protocol. Briefly, cells were plated in 24-well flat-bottom plates and treated with gemcitabine (5 μM) for 36 h. Cells were fixed in 4% paraformaldehyde at 4°C for 30 min, permeabilised in 0.1% Triton X-100, and labelled with fluorescein-12-dUTP using terminal deoxynucleotidyl transferase. The localised green fluorescence of apoptotic cells from the fluorescein-12-dUTP was detected by fluorescence microscopy (Axiovert 100M, Zeiss, Oberkochen, Germany).

### Xenografted tumor model and staining

BALB/c-nu mice (5–6 weeks old, 18–20 g) were purchased from the Experimental Animal Center of the Guangzhou University of Chinese Medicine and housed in barrier facilities on a 12-h light/dark cycle. The Institutional Animal Care and Use Committee of Sun Yat-sen University approved all experimental procedures. The mice were randomly assigned to groups (*n* = 8/group). The mice in groups were inoculated subcutaneously with PANC-1 cells (5 × 10^6^) in the left dorsal flank, and two weeks later, injected intratumorally with 100 μL miR-181c mimic, mimic control, antagomiR-181c control or antagomiR control (diluted in PBS at 2 mg/mL) three times per week for 4 weeks, combining with intraperitoneal injection of gemcitabine (50 mg/kg) weekly. Tumors were examined every 4 days; length, width, and thickness were measured with callipers, and tumor volumes were calculated. Tumor volume was calculated using the equation (L × W^2^)/2. On day 42, tumors were detected by an IVIS imagining system (Caliper), then animals were euthanized, tumors were excised, weighed and paraffin-embedded. Apoptotic index was measured by percentage of TUNEL-positive cells.

### Luciferase assay

Cells (4 × 10^4^) were seeded in triplicate in 24-well plates and cultured for 24 h. Cells were transfected with 100 ng TEAD reporter luciferase plasmid [27], or pGL3-MST1-3′UTR, or pGL3-LATS2-3′UTR, or pGL3-MOB1-3′UTR, or pGL3-SAV1-3′UTR luciferase plasmid, plus 5 ng pRL-TK Renilla plasmid (Promega) using Lipofectamine 2000 (Invitrogen) according to the manufacturer's recommendation. Luciferase and Renilla signals were measured 36 h after transfection using a Dual Luciferase Reporter Assay Kit (Promega) according to the manufacturer's protocol.

### Nuclear/cytoplasmic fractionation

Cells were washed with cold PBS and resuspended in buffer containing 10 mM HEPES (pH 7.8), 10 mM KCl, 0.1 mM EDTA, 1 mM Na_3_VO_4_, 1 mM DTT, 1:500 protease inhibitors (Sigma-Aldrich), and 0.2 mM PMSF, and incubated on ice for 15 min. Detergent was added and cells were vortexed for 10 s at the highest setting. Nuclei and the supernatant were separated by centrifugation at 4°C. Nuclei were resuspended in buffer containing 20 mM HEPES (pH 7.8), 0.4 M NaCl, 1 mM EDTA, 1 mM Na_3_VO_4_, 1 mM DTT, and 1:500 protease inhibitors and incubated on ice for 15 min. Nuclear extracts were collected by centrifugation at 14,000 × *g* for 10 min at 4°C.

### miRNP immunoprecipitation

Cells were co-transfected with HA-Ago1 together with 100 nM miR-181c, followed by HA-Ago1 immunoprecipitation using HA-antibody. Real-time PCR analysis of the IP material was used to test the association of the mRNA of MST1, LATS2, MOB1 and SAV1 with the RISC complex.

### Statistical analysis

All statistical analyses were carried out using SPSS statistical software (SPSS Inc., Chicago, IL, USA). Survival curves were plotted using the Kaplan–Meier method and compared by log-rank test. The 2-tailed Student's *t*-test was used to evaluate the significance of differences between two groups of data in all pertinent experiments. *P* < 0.05 was considered significant.

**Sequences of primers and siRNAs are provided in the supporting information.**

## SUPPLEMENTARY MATERIALS AND METHODS FIGURES AND TABLES


