# Supplementary material for: Upregulation of miR-181c contributes to chemoresistance in pancreatic cancer by inactivating the Hippo signaling pathway
Source: Oncotarget. 2015 Oct 26;6(42):44466–79. doi: 10.18632/oncotarget.6298 (PMC4792569; doi:10.18632/oncotarget.6298)
Supplement: Supplementary file 1 [file oncotarget-06-44466-s001.pdf]

# SUPPLEMENTARY MATERIALS AND METHODS

## Primers and siRNAs

**Real-time PCR primers:**

CTGF-up: 5′-CAGGCTAGAGAAGCAGAGCC-3′;
CTGF-dn: 5′-TGGAGATTTTGGGAGTACGG-3′;
BIRC5-up: 5′-TTGGTGAATTTTTGAAACTGGA-3′;
BIRC5-dn: 5′-CTTTCTCCGCAGTTTCCTCA-3′;
BCL2L1-up: 5′-TTCAGTGACCTGACATCCCA-3′;
BCL2L1-dn: 5′-CTGCTGCATTGTTCCCATAG-3′;
GAPDH-up: 5′-ATTCCACCCATGGCAAATTC-3′;
GAPDH-dn: 5′-TGGGATTTCCATTGATGACAAG-3′.

**miRNP primers:**

GAPDH-up: 5′-TCCCCCACCACACTGAATCT-3′;
GAPDH-dn: 5′-GCCCCTCCCCTCTTCAAG-3′;
MST1-up: 5′-GTGGCTAGAATATGGCAGAG-3′;
MST1-dn: 5′-GTAGATTGGGCAGGAACA-3′;
LATS2-up: 5′-ATATCTAGGCAACAACAC-3′;
LATS2-dn: 5′-AATAGCGAGAATACTGAC-3′;
MOB1-up: 5′-CCGATGGATTGGTTTCTG-3′;
MOB1-dn: 5′-TTGTTTCAAGCCCTGTTC-3′;
SAV1-up: 5′-ATTGTCTGGAAAGGGATG-3′;
SAV1-dn: 5′-GCTGGGATTACAGGAGTG-3′;

**siRNA sequences:**

YAP siRNA: 5′-CAGGUGAUACUAUCAACCAAA-3′;
TAZ siRNA: 5′-CCUGCCGGAGUCUUUCUUUAA-3′;
MST1 siRNA: 5′-CCGGCCAGAUUGUUGCUAUUA-3′;
LATS2 siRNA: 5′-CUACUCGCCAUACGCCUUUAA-3′.

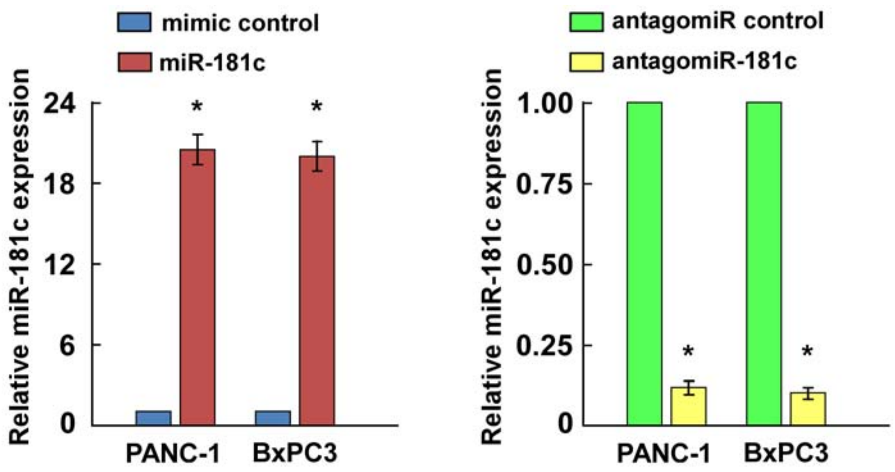


**Supplementary Figure S1: Real-time PCR analysis of miR-181c expression in PANC-1 and BxPC3 cells transfected with miR-181c mimic or antagomiR-181c as compared to the controls.** Transcript levels were normalized by *U6* expression. Error bars represent the mean ± s.d. of three independent experiments. *$P < 0.05$.

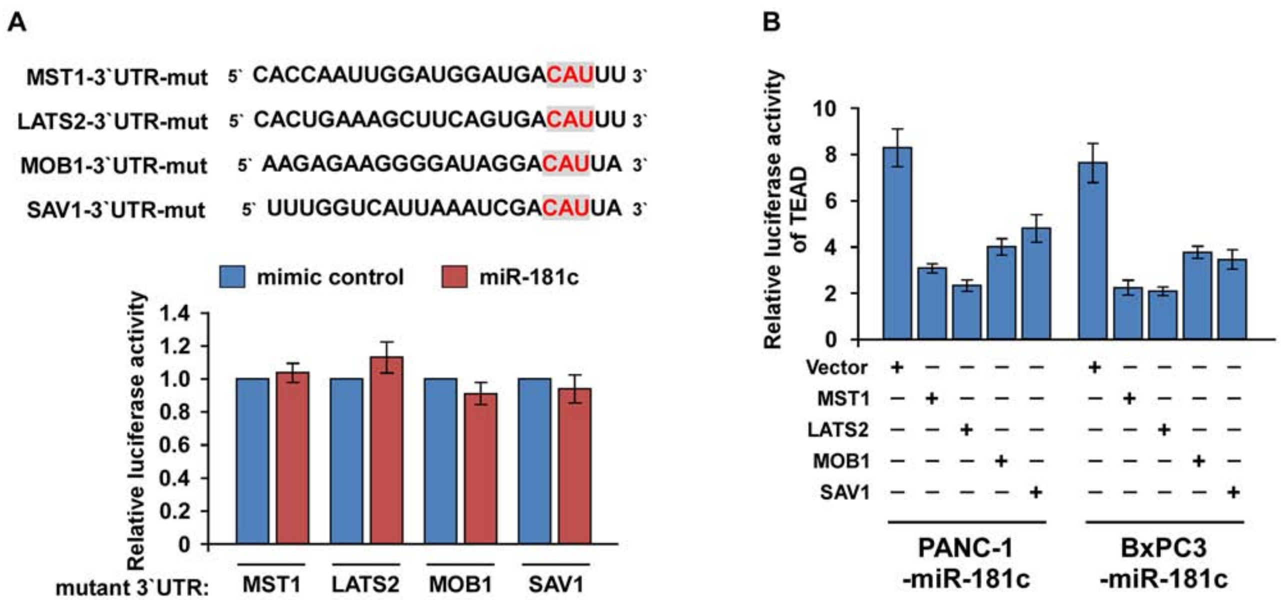


**Supplementary Figure S2: A. Ectopic expression of the miR-181c did not exhibit repressive effects on the reporter activities driven by the mutant 3′UTRs of these transcripts within miR-181c–binding seed regions. B.** Individual overexpression of MST1, LATS2, MOB1 or SAV1 potently inhibited the TEAD activity in miR-181c-overexpressing cells.

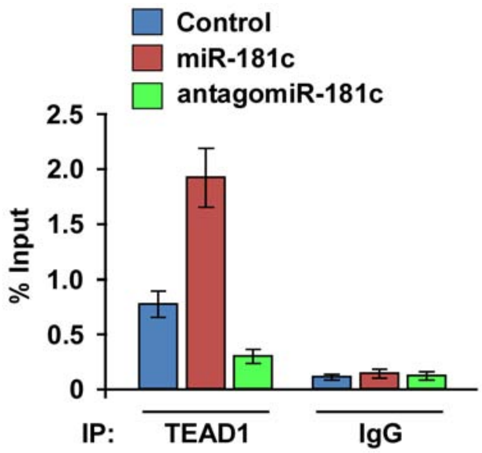


**Supplementary Figure S3: Chip assay showing that overexpression of miR-181c increased, while silencing miR-181c reduced the binding capability of TEAD1 with CTGF promoter.**

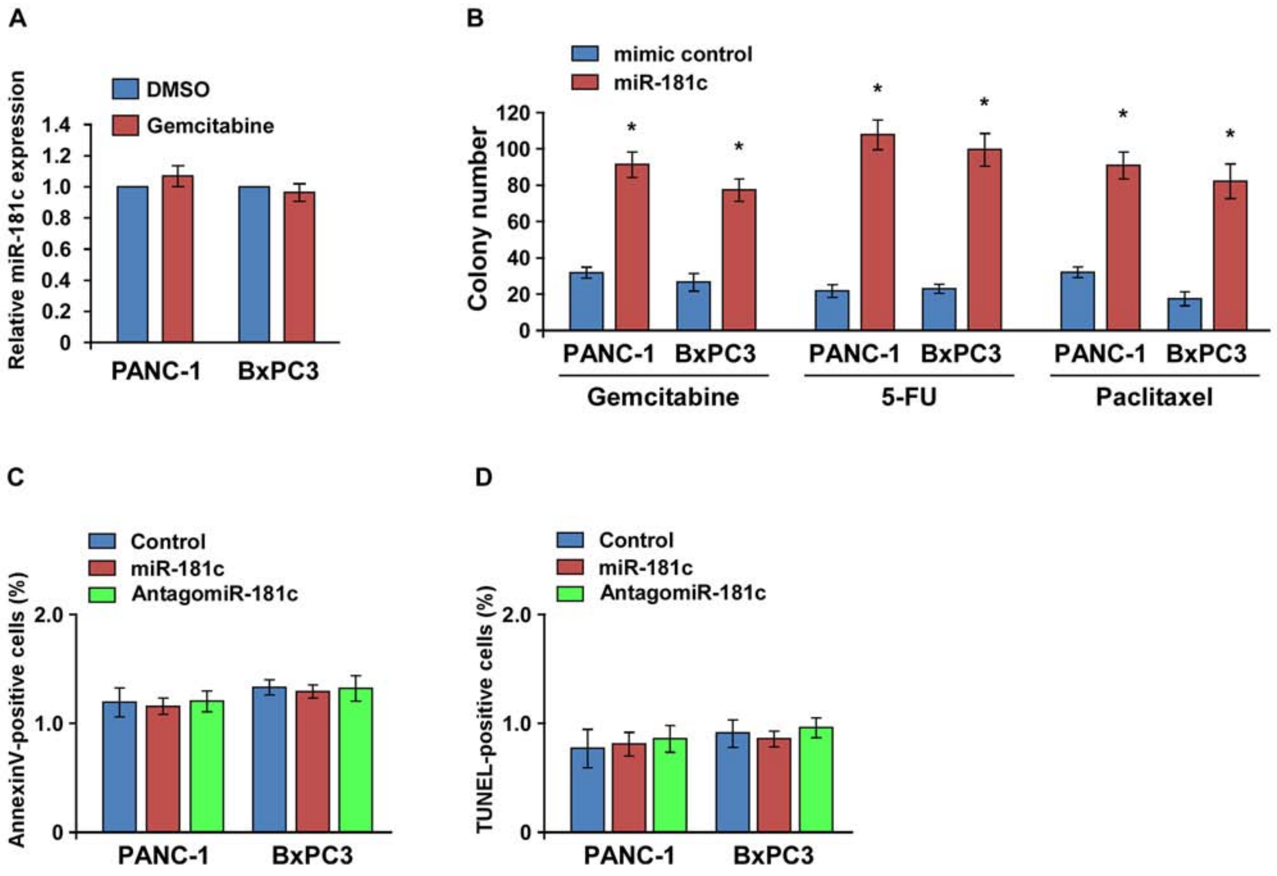


**Supplementary Figure S4: A. Real-time PCR analysis of miR-181c expression under gemcitabine treatment.** Transcript levels were normalized to *U6* expression. **B.** Quantification of crystal violet–stained PANC-1 and BxPC3 cell colonies overexpressing miR-181c in the presence of gemcitabine (5 μM), 5-FU (5 μM), or paclitaxel (10 μM). **C** and **D.** Annexin V-FITC/PI staining assay (C) and TUNEL analysis (D) revealed that neither overexpression of miR-181c nor silencing of miR-181c had an effect on the apoptotic percentage of PANC-1 and BxPC3 cell lines without chemo-drug treatment. Error bars represent the mean ± s.d. of three independent experiments. *$P < 0.05$.

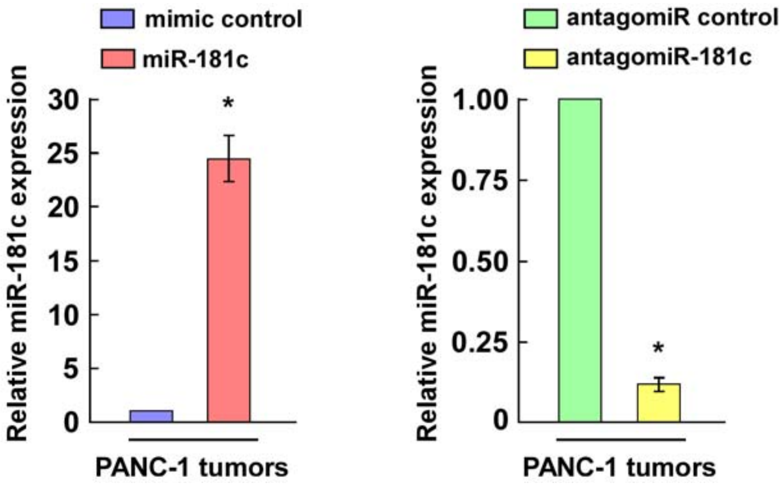


**Supplementary Figure S5: Real-time PCR analysis of miR-181c expression in PANC-1 xenograft tumors injected with miR-181c mimic or antagomiR-181c as compared to controls.** Transcript levels were normalized by *U6* expression. Error bars represent the mean ± s.d. of three independent experiments. *$P < 0.05$.

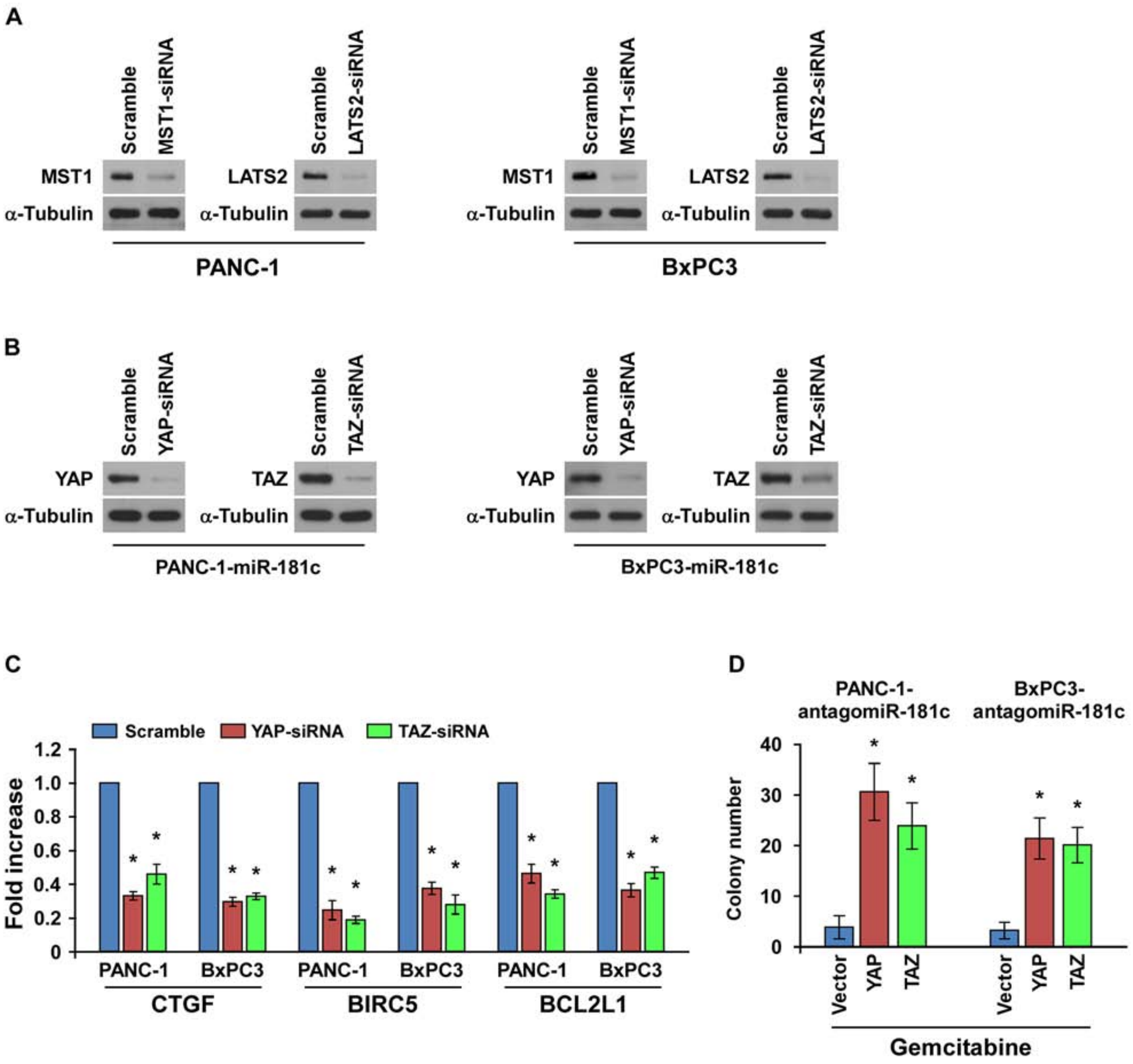


**Supplementary Figure S6: A. MST1 or LATS2 was silenced by siRNA transfection in pancreatic cancer cell lines, and their expression levels were examined by western blotting.** α-Tubulin served as the loading control. **B.** YAP or TAZ was silenced by siRNA transfection in miR-181–overexpressing cells, and their expression levels were examined by western blotting. α-Tubulin served as the loading control. **C.** Real-time PCR analysis revealed that the stimulatory effect of miR-181c on the expression of CTGF, BIRC5 and BCL2L1 were inhibited by YAP or TAZ silencing. **D.** Colony formation assay showed that overexpression of either YAP or TAZ significantly rendered resistance of miR-181c-silenced cells to gemcitabine. Error bars represent the mean ± s.d. of three independent experiments. $*P < 0.05$.

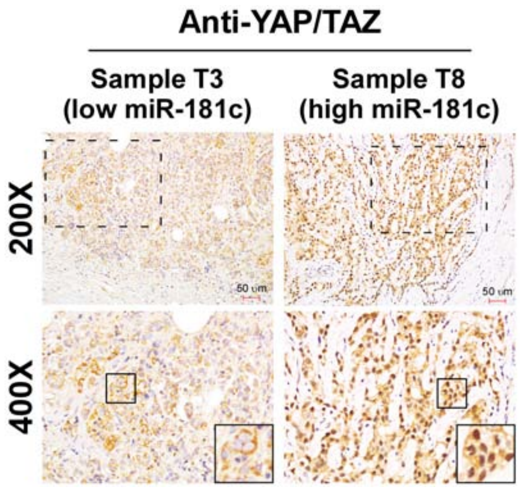


**Supplementary Figure S7: IHC staining of YAP/TAZ protein in pancreatic tissues.** Two representative cases (T3 with low miR-181c, T8 with high miR-181c) are shown. The results revealed that nuclear YAP/TAZ was strongly expressed in pancreatic cancer cells but not in the surrounding stromal cells, and correlated with miR-181c expression.

**Supplementary Table S1: Clinicopathological characteristics of studied patients and expression of miR-181c in pancreatic cancer**

| Factor | NO. | (%) |
|---|---|---|
| **Age (years)** | | |
| ≥60 | 52 | 41.9 |
| <60 | 72 | 58.1 |
| **Sex** | | |
| male | 84 | 67.7 |
| female | 40 | 32.3 |
| **Histological differentiation** | | |
| Grade 1/2 | 91 | 73.4 |
| Grade 3/4 | 33 | 26.6 |
| **T stage** | | |
| T1-2 | 82 | 66.1 |
| T3 | 42 | 33.9 |
| **N stage** | | |
| N0 | 69 | 55.6 |
| N1 | 55 | 44.4 |
| **M stage** | | |
| No | 46 | 37.1 |
| Yes | 78 | 62.9 |
| **Expression of miR-181c** | | |
| Low expression | 62 | 50.0 |
| High expression | 62 | 50.0 |

**Supplementary Table S2: Correlation between the clinicopathological features and expression of miR-181c**

| Patient characteristics | | miR-181c expression | | *P* value |
|---|---|---|---|---|
| | | Low | High | |
| **Age (years)** | ≥60 | 31 | 21 | 0.069 |
| | <60 | 31 | 41 | |
| **Sex** | male | 44 | 40 | 0.442 |
| | female | 18 | 22 | |
| **Histological differentiation** | Grade 1/2 | 52 | 39 | 0.008 |
| | Grade 3 | 10 | 23 | |
| **T Stage** | T1-2 | 48 | 34 | 0.008 |
| | T3 | 14 | 28 | |
| **N Stage** | N0 | 43 | 26 | 0.002 |
| | N1 | 19 | 36 | |
| **M Stage** | No | 31 | 15 | 0.003 |
| | Yes | 31 | 47 | |
